# Glucose Regulation of Load‐Induced mTOR Signaling and ER Stress in Mammalian Heart

**DOI:** 10.1161/JAHA.113.004796

**Published:** 2013-06-21

**Authors:** Shiraj Sen, Bijoy K. Kundu, Henry Cheng‐Ju Wu, S. Shahrukh Hashmi, Patrick Guthrie, Landon W. Locke, R. Jack Roy, G. Paul Matherne, Stuart S. Berr, Matthew Terwelp, Brian Scott, Sylvia Carranza, O. Howard Frazier, David K. Glover, Wolfgang H. Dillmann, Michael J. Gambello, Mark L. Entman, Heinrich Taegtmeyer

**Affiliations:** 1 Division of Cardiology, Department of Internal Medicine, The University of Texas Medical School at Houston, Houston, TX (S.S., H.C.J.W., P.G., M.T., H.T.); 2 Department of Radiology and Medical Imaging, University of Virginia, Charlottesville, VA (B.K.K., J.R., S.S.B.); 3 Department of Pediatrics, Pediatric Research Center, The University of Texas Medical School at Houston, Houston, TX (S.H.); 4 Department of Biomedical Engineering, University of Virginia, Charlottesville, VA (L.W.L., S.S.B.); 5 Department of Pediatrics, University of Virginia, Charlottesville, VA (P.M.); 6 Division of Endocrinology and Metabolism, Department of Medicine, University of California at San Diego, La Jolla, CA (B.S., W.H.D.); 7 Texas Heart Institute at St. Luke's Episcopal Hospital, Houston, TX (S.C., H.F.); 8 Cardiovascular Division, Department of Medicine, University of Virginia, Charlottesville, VA (D.K.G.); 9 Department of Human Genetics, Emory University School of Medicine, Atlanta, GA (M.J.G.); 10 Department of Medicine, Baylor College of Medicine, Houston, TX (M.L.E.)

**Keywords:** ER stress, glucose, hypertrophy, metabolism, mTOR

## Abstract

**Background:**

Changes in energy substrate metabolism are first responders to hemodynamic stress in the heart. We have previously shown that hexose‐6‐phosphate levels regulate mammalian target of rapamycin (mTOR) activation in response to insulin. We now tested the hypothesis that inotropic stimulation and increased afterload also regulate mTOR activation via glucose 6‐phosphate (G6P) accumulation.

**Methods and Results:**

We subjected the working rat heart *ex vivo* to a high workload in the presence of different energy‐providing substrates including glucose, glucose analogues, and noncarbohydrate substrates. We observed an association between G6P accumulation, mTOR activation, endoplasmic reticulum (ER) stress, and impaired contractile function, all of which were prevented by pretreating animals with rapamycin (mTOR inhibition) or metformin (AMPK activation). The histone deacetylase inhibitor 4‐phenylbutyrate, which relieves ER stress, also improved contractile function. In contrast, adding the glucose analogue 2‐deoxy‐d‐glucose, which is phosphorylated but not further metabolized, to the perfusate resulted in mTOR activation and contractile dysfunction. Next we tested our hypothesis in vivo by transverse aortic constriction in mice. Using a micro‐PET system, we observed enhanced glucose tracer analog uptake and contractile dysfunction preceding dilatation of the left ventricle. In contrast, in hearts overexpressing SERCA2a, ER stress was reduced and contractile function was preserved with hypertrophy. Finally, we examined failing human hearts and found that mechanical unloading decreased G6P levels and ER stress markers.

**Conclusions:**

We propose that glucose metabolic changes precede and regulate functional (and possibly also structural) remodeling of the heart. We implicate a critical role for G6P in load‐induced mTOR activation and ER stress.

## Introduction

Increases in the rates of energy substrate metabolism are the first responders to hemodynamic stress in the heart.^1^ When subjected to a sustained increase in pressure, hearts remodel both metabolically and structurally. Metabolically, hearts increase their reliance on carbohydrates for energy provision.^1–2^ Structurally, hearts hypertrophy.^3^ In independent studies we have previously demonstrated that the footprints of metabolic remodeling precede structural remodeling of the heart in hypertension and that activation of the insulin signaling pathway downstream of Akt requires hexose‐6‐phosphate.^4–5^ We now asked: Could the metabolic remodeling process also regulate signaling pathways of structural remodeling in the heart?

Remodeling is to some extent driven by the mammalian target of rapamycin (mTOR), a regulator of myocardial protein synthesis that is downstream of Akt in the insulin signaling pathway. mTOR kinase nucleates 2 major protein complexes—mTOR complex 1 (mTORC1) and mTOR complex 2 (mTORC2). Under unstressed conditions, mTORC1 activity in the heart is inhibited by the tuberous sclerosis complex (TSC), composed of hamartin (TSC1) and tuberin (TSC2). Phosphorylation of a number of serine and threonine residues on TSC2 relieves TSC's inhibition on mTORC1.^6–7^ Activated mTORC1 triggers protein synthesis and cardiac growth by phosphorylating p70S6 kinase (p70S6K) and 4E‐binding protein 1 (4EBP1) to promote ribosomal biogenesis and CAP‐dependent protein translation, respectively.

It is already known that mTORC1 plays a critical role in promoting cardiac growth in response to increased workload^8^ and that mTORC1 is activated by nutrients and growth factors.^9^ Whether inotropic mTORC1 activation is regulated by changes in energy substrate metabolism that occur in response to increased workload is unknown, however. We therefore investigated the functional and structural consequences of glucose‐mediated mTOR signaling in the stressed heart and discovered that it is both deleterious and preventable.

We also investigated whether sustained mTORC1 activation exhausts the capacity of the endoplasmic reticulum (ER) to fold proteins correctly, induces ER stress,^10^ and leads to contractile dysfunction in both rodent and human models of heart failure.^11^ The results suggest that dysregulated glucose metabolism mediates load‐induced contractile dysfunction by activating mTOR and inducing ER stress. We then went on to show the rescue effects of pharmacologic interventions (rapamycin, metformin, or 4‐PBA) and genetic manipulations (SERCA2a overexpression) on cardiac glucose metabolism and contractile function. Finally, we examined the effects of load reduction in failing human hearts after insertion of a left ventricular assist device and show that mechanical unloading reduces markers of dysregulated glucose metabolism and ER stress.

## Methods

To determine whether enhanced glucose uptake regulates mTOR activation at increased workload, we first tested the hypothesis *ex vivo*. The hearts were obtained from 4 groups of animals: (1) rats pretreated with vehicle for 7 days before *ex vivo* perfusion with noncarbohydrate substrates; (2) rats pretreated with vehicle for 7 days before *ex vivo* perfusion with glucose; (3) rats pretreated with mTOR inhibitor rapamycin for 7 days before *ex vivo* perfusion with NCS; (4) rats pretreated with the mTOR inhibitor rapamycin for 7 days before *ex vivo* perfusion with glucose. Table 1 lists the perfusion protocols. Each of the experimental groups was then evaluated in normal workload conditions for 60 minutes or for 30 minutes at normal workload followed by increased workload conditions for another 30 minutes. Hearts were freeze‐clamped at the end of the protocol.

**Table 1.** Ex Vivo Perfusion Protocols Used to Study Glucose Activation of mTOR Signaling at High Workload

Group	Protocol	Additions to Buffer
Noncarbohydrate substrates (NCS)Perfused	A	Propionate (2 mmol/L)+acetoacetate (5 mmol/L)
	B	No substrate (15 minutes of glycogen depletion) followed by propionate (2 mmol/L)+acetoacetate (5 mmol/L)
Glucose perfused	A	Glucose (5 mmol/L)
	B	Glucose (5 mmol/L)
	B	Glucose (5 mmol/L)*
	B	Glucose (5 mmol/L)*
	B	Glucose (5 mmol/L)+metformin (5, 7.5, or 10 mmol/L)
	B	Glucose (5 mmol/L)+4‐phenylbutyrate (10 mmol/L)
Glucose analogue perfused	A	2 Deoyx‐d‐glucose (5 mmol/L)+propionate (2 mmol/L)+acetoacetate (5 mmol/L)
	B	2 Deoyx‐d‐glucose (5 mmol/L)+propionate (2 mmol/L)+acetoacetate (5 mmol/L)
	A	3‐O‐methylglucose (5 mmol/L)+propionate (2 mmol/L)+acetoacetate (5 mmol/L)
	B	3‐O‐methylglucose (5 mmol/L)+propionate (2 mmol/L)+acetoacetate (5 mmol/L)
Mixed substrates perfused	A	Glucose (5 mmol/L)+oleate (0.4 mmol/L)+propionate (2 mmol/L)+acetoacetate (5 mmol/L)
	B	Glucose (5 mmol/L)+oleate (0.4 mmol/L)+propionate (2 mmol/L)+acetoacetate (5 mmol/L)

mTOR indicates mammalian target of rapamycin; AMPK, AMP kinase. A, hearts were perfused at normal workload (15 cm H_2_O preload, 70 cm H_2_O afterload) for 60 minutes; B, hearts were perfused at normal workload for 30 minutes, followed by high workload (15 cm H_2_O preload, 100 cm H_2_O afterload, plus 0.1 μmol/L epinephrine) for another 30 minutes.

* Group was pretreated with mTOR inhibitor rapamycin (4 mg/kg per day PO) for 7 days before perfusion.

* Group was pretreated with AMPK activator metformin (250 mg/kg per day IP or 500 mg/kg per day IP) for 7 days before perfusion.

### Rationale for Animal Models Used and Overall Strategy

The structural and functional response to increased workload has been extensively studied in the murine transverse aortic constriction (TAC) model, first validated by Rockman et al.^12^ How the metabolic response to increased workload regulates these changes has yet to be investigated. To systematically answer this question, we used 3 models. First, we used the isolated working rat heart *ex vivo* to elucidate the mechanisms for the observed phenomena in vivo. The isolated working rat heart permitted a dynamic minute‐by‐minute assessment of metabolism and function and allowed for assessment of intracellular metabolites and activation of relevant signaling pathways at the end of the perfusion. The *ex vivo* isolated working heart model was also chosen because it gave us complete control over workload, substrate concentration, and hormone supply. Second, we optimized in vivo 2‐deoxy, 2[^18^F]fluorodeoxy‐glucose positron emission tomography (FDG‐PET) and magnetic resonance imaging (MRI) to simultaneously image changes in metabolism, function, and structure in mice subjected to TAC for up to 4 weeks. In this model we established that metabolic remodeling (enhanced glucose uptake) and contractile dysfunction precede structural remodeling (growth and dilation of the left ventricle). In addition, a set of mice underwent in vivo aortic banding for molecular and metabolite analyses. ER stress marker was examined in the SERCA2a overexpressing mouse genetic model to correlate with functional rescue of cardiac power when challenged with TAC. Third, we analyzed left ventricular tissue from failing human heart muscle before and after mechanical unloading with a left ventricular assist device to correlate our findings to the failing human heart.

### Experimental Groups

The experimental groups are listed in Table 1. The animals received standard laboratory chow and water ad libitum. A subset of rats received either rapamycin (4 mg/kg per day) or vehicle control (propylene glycol) by oral gavage for 7 days before experimentation (Table 1). A second subset received either metformin (250 or 500 mg/kg per day) or vehicle control (saline) intraperitoneally for 7 days before experimentation. On day 7, animals were anesthetized with chloral hydrate (300 mg/kg per day) and anticoagulated with heparin (200 U) immediately before their hearts were isolated and perfused in the working mode as described previously.^13^ A third subset of untreated rats hearts were isolated and perfused with either metformin (10, 5, 1 mmol/L) or sodium 4‐phenylbutyrate (10 mmol/L) added directly to the perfusion buffer. Substrate concentrations and workload are listed in (Table 1).

### Isolated Working Rat Heart Perfusions

To assess rates of myocardial glucose metabolism in response to increased workload, male Sprague‐Dawley rats (387±11 g) were obtained from Harlan Laboratories (Indianapolis, IN) and housed in the Animal Care Center of the University of Texas Medical School at Houston under controlled conditions (23±1°C; 12‐hour light/12‐hour dark cycle).

Briefly, hearts were perfused as working hearts^13^ in the presence or absence of glucose (5 mmol/L) plus 0.05 μCi/L [2‐^3^H]‐glucose to measure rates of glucose uptake and 20 μCi/L [U‐^14^C]‐glucose to measure rates of glucose oxidation. In the absence of glucose, sodium propionate (2 mmol/L) plus lithium acetoacetate (5 mmol/L) were used as a noncarbohydrate substrate (NCS) control. Together these substrates fuel the Krebs cycle without generating any upstream metabolites^14^ and sustain cardiac work in the absence of glucose and in the presence of nonmetabolizable glucose analogues.^15^ Hearts perfused with NCS were first perfused substrate‐free for 15 minutes to deplete endogenous glycogen stores.^1^ Under steady‐state conditions, hearts were perfused at a normal (physiological) workload (afterload set to 100 cm H_2_O) for 60 minutes (protocol A). Hearts subjected to a high workload were perfused for 30 minutes at normal workload followed by 30 minutes of perfusion at increased workload (afterload raised to 140 cm H_2_O plus 1 μmol/L epinephrine bitartrate added to the perfusate; protocol B). Left atrial pressure was 15 cm H_2_O in each perfusion protocol. At 60 minutes the beating hearts were freeze‐clamped with aluminum tongs cooled in liquid nitrogen. A portion of frozen heart tissue was weighed and dried to constant weight. The remainder was stored at −80°C for further analyses.

### Cardiac Power, Oxygen Consumption, Metabolic Rates, Metabolite Concentrations

In *ex vivo* isolated working hearts, coronary and aortic flows were recorded every 5 minutes, and aortic pressures were recorded continuously with a 3F Millar pressure transducer (Millar Instruments, Houston, TX) in sidearm to the aortic cannula linked to a physiologic recorder (Gould Model 2400S, Gould Instruments, Cleveland, OH). Cardiac power (in milliwatts) was calculated as the product of cardiac output (coronary plus aortic flow, m^3^/s) and mean aortic pressure (in pascals).

Myocardial oxygen consumption and rates of glucose uptake and oxidation were determined as previously described.^1,16^ ATP, ADP, and AMP levels were quantified in freeze‐clamped hearts as described previously.^17^ Glucose 6‐phosphate levels were measured by spectrophotometric enzymatic analysis using glucose 6‐phosphate dehydrogenase coupled to NADPH production with extinction at 340 nm.^18^

### PET and MRI Imaging

Male C57BL/6 mice (8 to 9 weeks of age) obtained from Charles River Laboratories (Raleigh, NC) were subjected to sham operation or transverse aortic constriction to induce pressure‐overload hypertrophy by methods previously described.^19^ All mice were housed in a 12‐hour light/12‐hour dark cycle and given standard laboratory chow and water ad libitum. Prior to imaging, animals were fasted overnight with access to water. All experiments were performed in compliance with the Guide for the Care and Use of Laboratory Animals, published by the National Institutes of Health, and were conducted under protocols approved by the Animal Care and Use Committee at the University of Virginia.

Left ventricular pressure measurements were performed using a Mikro‐tip Catheter Transducer (Model SPR‐671, size 1.4F; Millar Instruments Inc, Houston, TX) connected to Millar and PowerLab hardware and ADI Software (AD Instruments Inc, Colorado Springs, CO). Animals were anesthetized with 3% isoflurane and maintained at 1% to 2% while the catheter was placed in the right carotid artery under stereomagnification and advanced through the aorta so that the tip was positioned in the left ventricle. Heart rate and cardiac pressure were continuously monitored throughout. Body temperature was maintained at 37°C with a warming blanket.

FDG‐PET and MRI were sequentially performed in anesthetized animals. PET imaging was performed in a Focus 120 micro PET scanner (Siemens Molecular Imaging Inc, Knoxville, TN) to assess changes in metabolism. The protocol was initiated by injecting 150 to 200 μL of 18.5‐37 MBq FDG solution intravenously over a period of 1 minute. Myocardial glucose uptake was assessed using FDG (IBA Molecular, Sterling, VA) at baseline, 1 day, 2 weeks, and 4 weeks after surgery. The net FDG influx constant, K_i_ (mL/min per gram),^20^ was measured over a period of 4 weeks. Determination of K_i_ is considered a robust index of glucose transport and phosphorylation.^21^ Therefore, our present work focused on determination of K_i_ with partial volume and attenuation corrections in vivo. Throughout the procedure, heart rate, respiration, and body temperature were recorded with a physiological recorder (model 1025L; SA Instruments, Inc, Stony Brook, NY), and the ECG was monitored (Blue Sensor, Ambu Inc, Glen Burnie, MD). All the signals were sent to a single SA Instruments software interface, which was configured to trigger data acquisition based on the ECG. The SAI 1025L was also used to maintain the animal's core body temperature at 37°C.

Mice were also imaged using a 7T Bruker‐Siemens Clinscan MRI system for structural and functional analyses of the heart. After once again being anesthetized, each mouse was placed prone inside the radiofrequency coil, and the ECG leads were connected to an ECG monitoring module (SA Instruments, Inc, Stony Brook, NY). An ECG‐triggered 2‐dimensional cine gradient echo pulse sequence was used,^22^ with a slice thickness of 1 mm and a zero‐filled, in‐plane resolution of 100×100 μm^2^. During each session, the entire left ventricle (LV) was imaged with 6 to 7 contiguous short‐axis slices. The MR images were analyzed using the ARGUS software package (Siemens Medical Systems, Erlangen, Germany) for left ventricular end‐systolic volume, left ventricular end‐diastolic volume, and left ventricular ejection fraction was computed from the traced borders. Epicardial contours were also traced at the end‐diastolic and end‐systolic phases to compute LV mass.^23^ This was divided by the body weight to obtain the heart weight to body weight (HW/BW) ratios.

### Transgenic Mice Overexpressing Cardiac‐Specific Isoform of SERCA2a

Transgenic mice overexpressing rat SERCA2a (TG) in heart were produced as described before.^24^ Ascending aortic constriction was performed in both TG and age‐matched wild‐type mice to induce pressure‐overload left ventricular hypertrophy.^25^ Mice were studied 8 weeks after surgery. Cardiac function was assessed by M‐mode echocardiography. Subsequently, hearts were freeze‐clamped and stored at the temperature of liquid N_2_ before extraction at a later time. Sham‐operated mice were used as controls.

### Tissues From Failing Human Hearts

Cardiac tissue samples were obtained from 11 nondiabetic patients with idiopathic dilated cardiomyopathy (10 were male) referred to the Texas Heart Institute for heart transplantation and placed on left ventricular assist device (LVAD) support for a mean duration of 254±63 days. Tissue from the left ventricular apex was obtained during LVAD implantation and again during LVAD explantation (each patient went on to transplantation). Tissue samples were immediately frozen in liquid nitrogen and stored at −80°C for metabolic and molecular analyses at a later time. Table 2 provides further detail on the patients assessed in this study. The protocol was approved by the Committee for the Protection of Human Subjects of St. Luke's Episcopal Hospital in Houston, Texas, and of the University of Texas Medical School at Houston.

**Table 2.** Clinical Data: Samples From Failing Human Hearts

Sex (M/F)	10/1		
Ethnicity	5 White	5 African American	1 Hispanic
Age (y), mean±SEM	48±4 (19 to 67)		
LVEDD before LVAD, mean±SEM	75.33±4.40 mm		
LVEDD after LVAD, mean±SEM	55.67±3.22 mm		
EF before LVAD, mean±SEM	21±3.00%		

LVEDD indicates left ventricular end‐diastolic dimension; LVAD, left ventricular assist device; EF, ejection fraction.

### Western Blot Analyses

Protein was extracted from frozen heart tissue after homogenization (Polytron, Brinkman Instruments), and Western blot analyses were performed as previously described.^26^ Antibodies for PI3K, Akt 1/2, TSC2, mTOR, RAPTOR, p70S6K1, 4EBP1, AMP kinase (AMPK), acetyl CoA carboxylase (ACC), phospho‐PI3K p85 (Tyr458), phospho‐Akt1/2 (Ser473), phospho‐TSC2 (Ser939), phospho‐TSC2 (Ser1387), phospho‐mTOR (Ser2448), phospho‐p70S6K (Thr389), phospho‐4EBP1 (Thr70), phospho‐AMPKinase (Thr172), phospho‐ACC (Ser79), and phospho‐RAPTOR (Ser792) were obtained from Cell Signaling (Beverly, MA). Antibodies for glucose‐regulated protein 78 (GRP78), glucose‐regulated protein 94 (GRP94), and ER protein 72 (ERp72) were purchased from Enzo Life Sciences (Plymouth Meeting, PA), and GADD153 was purchased from Santa Cruz Biotechnology (Santa Cruz, CA). GAPDH was used to normalize for protein loading (Research Diagnostics, Flanders, NJ). Densitometry was performed with ImageJ program provided by the NIH. For clarity, densitometry values (mean±SEM) are presented for all blots collectively following the figures.

### RNA Extraction and qRTPCR

For transcriptional analysis, total RNA was extracted with TRI Reagent (Molecular Research Center, Cincinnati, OH) and treated for genomic DNA contamination with DNA‐*free* (Applied Biosystems/Ambion, Austin, TX). RNA concentration was measured with a NanoDrop 1000 Spectrophotometer (Thermo Scientific, Wilmington DE). Absolute quantification of transcripts was based on known amounts of synthetic DNA standard (Integrated DNA Technologies, Coralville, IA). Primer and probe sequences for GRP78, GRP94, and ERp72 have been previously published.^27^

### Statistical Analyses

The following is a summary of statistical methods used in preparing information shown in the accompanying figures.

**Figure 1.**
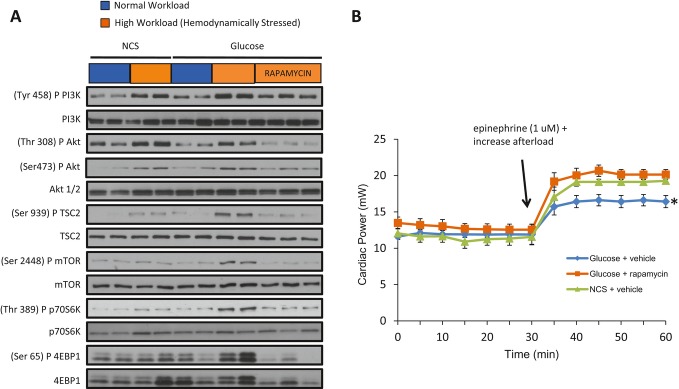
At high workload, glucose activates mTOR and impairs cardiac power in perfused rat hearts. Pretreatment of animals with rapamycin inhibits mTOR and rescues contractile function. A, Representative Western blots of the mTOR signaling pathway in hearts freeze‐clamped at the end of perfusion. High workload resulted in phosphorylation of PI3K and Akt in all hearts. For groups, protocols, and additions to the buffer, consult Table 1. However, phosphorylation of TSC2, mTOR, and p70S6K at high workload occurred only in the presence of glucose. Pretreating rats for 7 days with rapamycin (4 mg/kg per day) before perfusion of the heart resulted in decreased phosphorylation of Akt, TSC2, mTOR, and its downstream targets. B, At normal workload there was no difference in cardiac power among the 3 groups. At high workload the presence of the glucose substrate decreased cardiac power in hearts from vehicle‐treated rats. Pretreatment of rats with rapamycin for 7 days rescued cardiac performance in glucose‐perfused hearts. Data shown are mean±SEM; n=5 to 7 for each group. *P<0.05 (Mann–Whitney rank sum test) for hearts from vehicle‐treated rats perfused with glucose in comparison with hearts from vehicle‐treated rats perfused with NCS and those from rats pretreated with rapamycin and perfused with glucose at high workload. mTOR indicates mammalian target of rapamycin; TSC2, tuberin; NCS, noncarbohydrate substrate.

For Figure 1 we examined the effects of glucose on cardiac power in stressed hearts using *ex vivo* rat heart perfusions by comparing NCS+vehicle (n=6), glucose+vehicle (n=5), and glucose+rapamycin (n=5). Repeated measures for mice in a group were compared using a Friedman test separately for normal and high workload times. Comparisons of the paired median values for each animal within a group at normal and high workloads were performed using a Wilcoxon signed rank test. The median cardiac power for each animal at high workload was compared between groups using a Mann–Whitney rank sum test.

**Figure 2.**
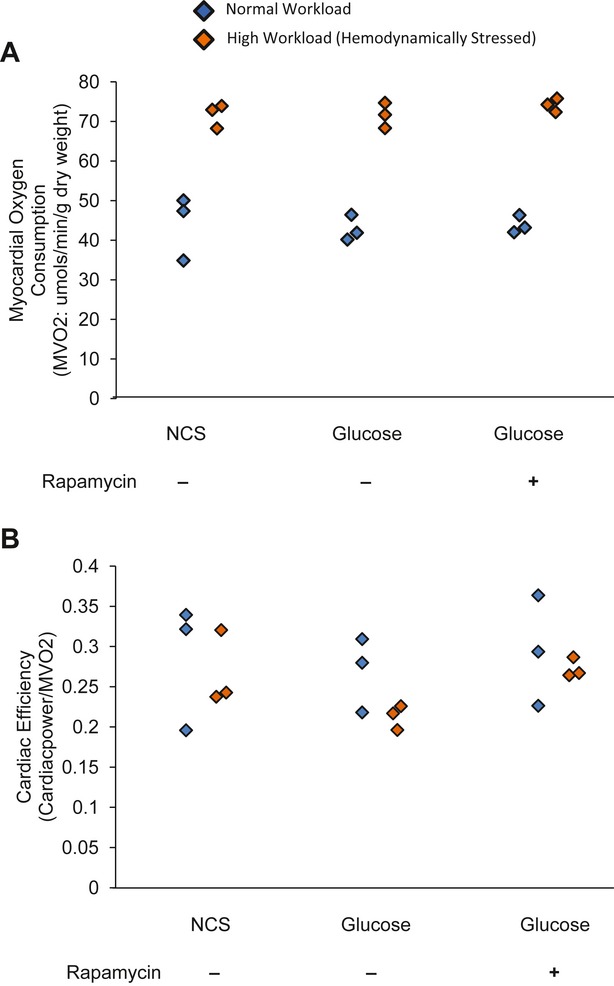
Pretreatment with rapamycin has no effect on cardiac efficiency in rat hearts perfused with glucose at high workload. A, Myocardial oxygen consumption (MVO_2_) at low and high workloads did not differ in hearts perfused with noncarbohydrate substrate (NCS) and in hearts from rats pretreated with vehicle or with rapamycin and perfused with glucose. B, Cardiac efficiency, as calculated by dividing cardiac power by MVO_2_, was also unchanged. The dot plots show individual measurements for each heart at 2 different workloads. Comparison within each group and between groups showed no significant difference (*P*>0.05).

For Figure 2 we examined the effects of glucose on cardiac efficiency in stressed hearts using *ex vivo* rat heart perfusions by comparing NCS+vehicle (n=3), glucose+vehicle (n=3), and glucose+rapamycin (n=3). Repeated measures for mice in a group were compared using a Friedman test separately for normal and high workload points. Comparisons of the paired median values for each animal within a group at normal and high workloads were performed using a Wilcoxon signed rank test. The median cardiac efficiency for each animal at high workload in the glucose+vehicle group was compared separately to the NCS+vehicle and glucose+rapamycin groups using a Mann–Whitney rank sum test.

**Figure 3.**
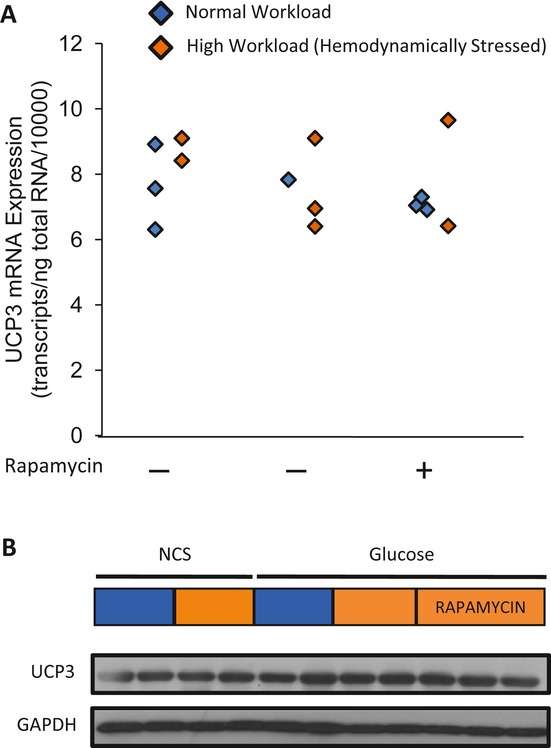
Uncoupling protein 3 (UCP3) mRNA and protein levels are not associated with G6P‐mediated mammalian target of rapamycin (mTOR) activation. A, Transcript analysis of *UCP3* gene expression in isolated working rat hearts. The dot plots show median values for n=2 to 3 rat hearts with 2 to 3 repeats per animal. *P*>0.05 for all comparisons based on Friedman test on repeated measurements of all hearts in each group compared with hearts perfused with noncarbohydrate substrate (NCS) at a normal workload. Neither workload nor glucose in the perfusate nor pretreatment of mice with rapamycin changed UCP3 mRNA expression at the end of perfusion. B, Compared with hearts perfused with NCS at a normal workload, neither workload, glucose, nor rapamycin pretreatment changed UCP3 protein levels.

For Figure 3 we examined the effects of glucose on mitochondrial efficiency in stressed hearts using ex vivo rat heart perfusions. We compared between repeated measures of NCS+vehicle (n=3), NCS+vehicle+workload (n=2), glucose+vehicle (n=1), glucose+vehicle+workload (n=3), glucose+rapamycin (n=3), and glucose+rapamycin+workload (n=2) using a Friedman test.

**Figure 4.**
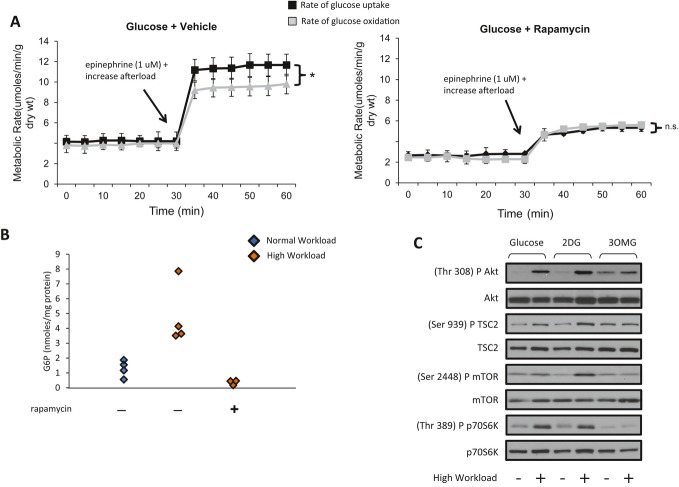
In response to high workload, rates of glucose uptake exceed rates of glucose oxidation, hexose 6‐phosphate accumulates, and mTOR is activated. A, Rates of glucose uptake exceeded rates of glucose oxidation in hearts from rats pretreated with either vehicle or rapamycin and perfused at normal and high workloads. Pretreating rats with rapamycin significantly reduced rates of both glucose uptake and oxidation (right). Data shown are mean±SEM; n=5 to 6 per group. *P=0.07 with Mann–Whitney rank sum test. B, Glucose 6‐phosphate (G6P) levels in freeze‐clamped hearts. Subjecting hearts to high workload *ex vivo* induced a 4‐fold increase in average G6P levels, which was not observed when rats were pretreated with rapamycin. Dot plots show G6P levels for each heart. Kruskal–Wallis test yielded overall *P*=0.012. C, To test the hypothesis that hexose‐6‐phosphate (and no other glucose metabolite) activates mTOR, we also perfused hearts with NCS plus 2‐deoxyglucose or 3‐O‐methylglucose (see text for details). Representative Western blots of Akt, TSC2, mTOR and p70S6K phosphorylation from hearts perfused with glucose (5 mmol/L) or NCS plus the glucose analogues 2‐deoxyglucose (2DG; 5 mmol/L), or 3‐O‐methylglucose (3OMG; 5 mmol/L) at either normal or high workload are shown. Glucose or 2DG, but not 3OMG, increased phosphorylation of TSC2, mTOR, and p70S6K at high workload. mTOR indicates mammalian target of rapamycin; TSC2, tuberin; NCS, noncarbohydrate substrate.

For Figure 4 we examined the effect of glucose on mTOR activation in ex vivo stressed hearts by comparing NCS+vehicle (n=6), glucose+vehicle (n=5), and glucose+rapamycin (n=5). Repeated measures for mice in a group were compared using a Friedman test separately for normal and high workload times. Comparisons of the paired median values for each animal within a group at normal and high workloads were performed using a Wilcoxon signed rank test. We also assessed the effects of mTOR modulation on G6P levels in *ex vivo* hearts by comparing glucose (n=4), glucose+workload (n=4), and glucose+workload+rapamycin (n=3). Median glucose uptake and glucose oxidation at high workload for glucose+vehicle and glucose+rapamycin groups were compared using a Mann–Whitney rank sum test. Comparisons of the G6P levels in glucose+normal workload (n=4), glucose+high workload with (n=3) or without rapamycin (n=4) were done using a Kruskal–Wallis test.

**Figure 5.**
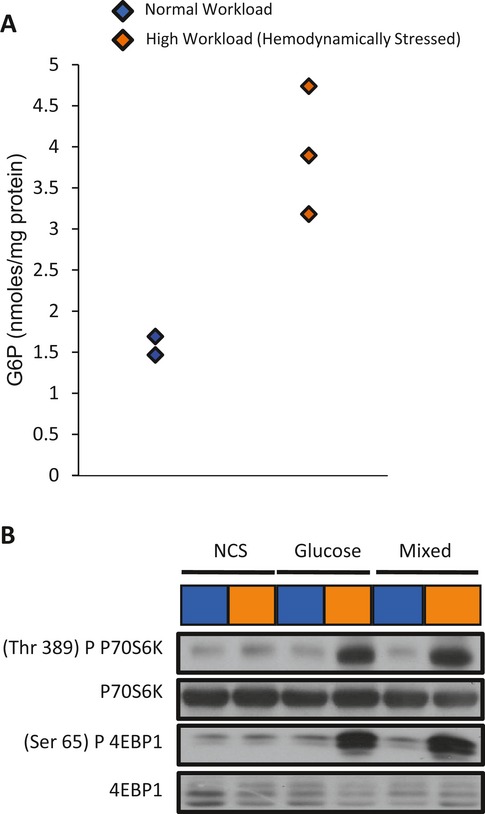
In the presence of mixed substrates (glucose, NCS, and oleate), G6P levels are increased and mTOR signaling is activated at high workload. A, G6P accumulates in hearts perfused with glucose, oleate, and NCS (see Table 1 for substrate concentrations). Dot plot shows G6P levels for each heart at the end of perfusion at low or high workload; *P*=0.083 using Mann–Whitney test. B, Representative Western blots showing phosphorylation of mTOR targets p70S6K and 4EBP1 in *ex vivo* working rat (see Table 1 for details). Both glucose and mixed substrates in the perfusate increased p70S6K and 4EBP1 phosphorylation. NCS indicates noncarbohydrate substrate; mTOR, mammalian target of rapamycin; G6P, glucose 6‐phosphate.

For Figure 5 we tested accumulation of G6P in stressed hearts under physiologic conditions by comparing *ex vivo* rat hearts perfused with either physiologic substrate with (n=3) or without an increased workload (n=2), using a Mann–Whitney test.

**Figure 6.**
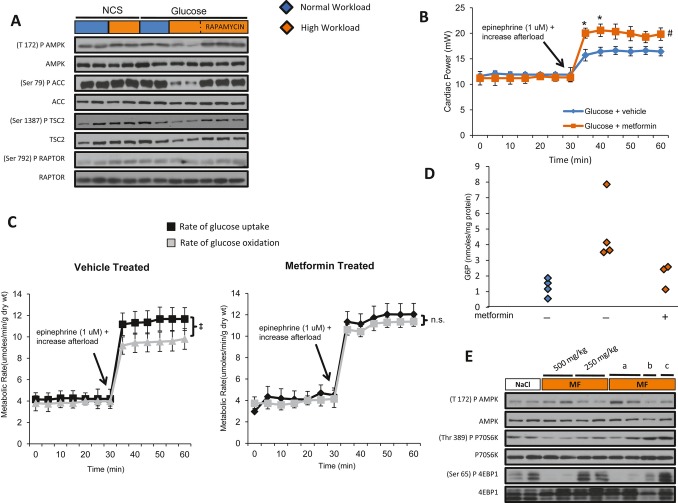
G6P‐dependent mTOR activation is associated with downregulation of AMPK. A, Representative Western blots demonstrate reduction in phosphorylation of AMPK (T172) and ACC (S79), its downstream target, in hearts perfused with glucose at high workload. B, Contractile performance in the working heart perfused with glucose in the presence and absence of metformin. Data shown are mean±SEM; n=5 to 7 for each group. Metformin improves cardiac power at high workload. *P<0.05, #*P*=0.08, using Mann–Whitney rank sum test. C, Rates of glucose uptake and oxidation by hearts from animals receiving vehicle or metformin pretreatment for 7 days (vehicle treated, metformin treated). Data shown are mean±SEM; n=5 to 6 per group. Metformin treatment did not change rates of glucose uptake and oxidation at normal workload. At high workload, pretreating animals with metformin corrected the mismatch between rates of glucose uptake and oxidation. ‡*P*=0.021 using Mann–Whitney rank sum test. D, G6P levels in hearts from rats receiving either vehicle or metformin for 7 days before perfusion of their hearts with glucose as the only substrate. Dot plots of G6P show individual measurements for each heart at normal and high workloads. G6P accumulation was reduced in stressed hearts perfused with glucose in hearts of animals pretreated with metformin. Kruskal–Wallis test yielded an overall *P*=0.012. E, Representative Western blots demonstrating increased AMPK phosphorylation and decreased p70S6K as well as 4EBP1 phosphorylation in hearts from animals pretreated for 7 days with metformin (500 or 250 mg/kg) and perfused with glucose at high workload or in hearts from untreated animals perfused with glucose plus metformin at high workload. The concentrations of metformin in the perfusate were (a) 10 mmol/L, (b) 7.5 mmol/L, or (c) 5 mmol/L. Also see Table 1 for experimental detail. mTOR indicates mammalian target of rapamycin; AMPK, AMP kinase; ACC, acetyl‐CoA carboxylase; G6P, glucose 6‐phophate; MF, metformin.

For Figure 6 we examined the effects of AMPK modulation on cardiac parameters *ex vivo* by comparing glucose+vehicle (n=5) and glucose+metformin (n=5). Repeated measures for mice in a group were compared using a Friedman test separately for normal and high workload times. Comparisons of the paired median values for each animal within a group at normal and high workloads were performed using a Wilcoxon signed rank test. Median glucose uptake and glucose oxidation at high workload for glucose+vehicle and glucose+metformin groups were compared using a Mann–Whitney rank sum test. We also assessed the effects of AMPK modulation on G6P levels in *ex vivo* hearts by comparing glucose (n=4), glucose+workload (n=4), and glucose+workload+metformin (n=3) with a Kruskal–Wallis test. Differences in cardiac power between normal and high workloads were assessed using a Friedman test. Median cardiac power per heart at high workload in the glucose+vehicle group was compared with the glucose+metformin group using a Mann–Whitney rank sum test.

**Figure 7.**
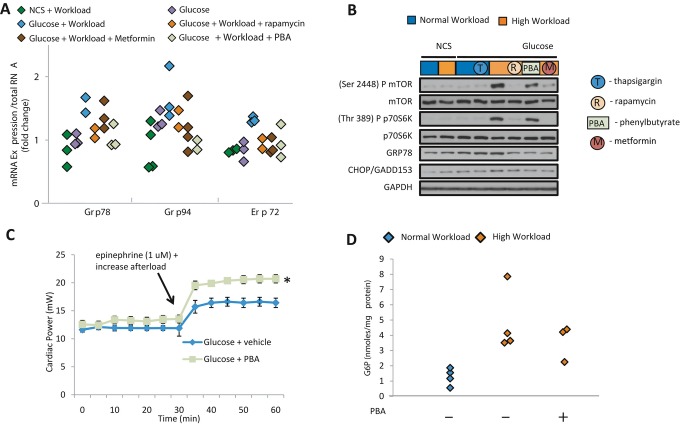
Relief of ER stress improves contractile function in hearts perfused with glucose at high workload. A, Transcript analysis of ER stress response chaperones in isolated working rat hearts. Dot plot shows measurement of transcript levels of each ER stress marker with different treatment (n=2 to 3 for each group, with 2 to 3 repeats per heart). Hearts perfused with glucose at high workload showed a nearly 2‐fold average increase in markers of ER stress, which was not observed when animals were pretreated with rapamycin or metformin or when phenylbutyrate (PBA) was added to the perfusate. For details on groups, protocols, and additions to the buffer, see Table 1. Friedman test for ER stress (GRP78, GRP94, and ERP72) are 0.26, 0.10, and 0.71, respectively. B, Representative Western blots of mTOR, p70S6K, GRP78, and CHOP. Rapamycin or metformin inhibited mTOR signaling and lowered GRP78 protein. Adding PBA to perfusate lowered GRP78 levels independent of changes in mTOR signaling. Thapsigargin, an inhibitor of SERCA2a and a direct stress inducer for the ER, served as control. Taken together, these data suggest that the induction of ER stress in glucose‐perfused hearts at high workload requires mTOR activation and can be prevented by systemic rapamycin or metformin pretreatment. C, Effect of addition of PBA (10 mmol/L) to the perfusate on contractile performance of hearts perfused with glucose. Data shown are mean±SEM (n=5 to 8 for each group). PBA improved cardiac power at high workload. *P<0.004 using Mann–Whitney rank sum test. D, Effect of PBA on G6P accumulation in isolated working rat hearts. Dot plots of G6P levels are shown. Kruskal–Wallis test yielded an overall *P*=0.012, but comparison of G6P levels at high workload with or without metformin treatment was not statistically significant (*P*>0.05) using a Mann–Whitney test. ER indicates endoplasmic reticulum; NCS, noncarbohydrate substrate; mTOR, mammalian target of rapamycin.

For Figure 7 levels of ER stress markers were interrogated in response to glucose and stress using *ex vivo* rat heart perfusions. The repeated measures of NCS+workload (n=3 to 4, 3 repeats per n), glucose (n=3, 3 repeats per n), glucose+workload (n=3, 2 to 3 repeats per n), glucose+workload+rapamycin (n=2, 2 to 3 repeats per n), glucose+workload+metformin (n=3 to 4, 3 repeats per n), and glucose+workload+PBA (n=2 to 3, 2 to 3 repeats per n) were compared using a Friedman test. Next, we assessed the effects of ER stress relief on cardiac power using *ex vivo* rat heart perfusions by comparing glucose+vehicle (n=5) and glucose+PBA (n=6). Repeated measures for mice in a group were compared using a Friedman test separately for normal and high workload points. Comparisons of the paired median values for each animal within a group at normal and high workloads were performed using a Wilcoxon signed rank test. Median cardiac power for each animal at high workload was compared between groups using a Mann–Whitney rank sum test. Finally, to see if ER stress relief also affected G6P accumulation, we compared glucose (n=4), glucose+workload (n=4), and glucose+workload+PBA (n=3) using a Kruskal–Wallis test.

**Figure 8.**
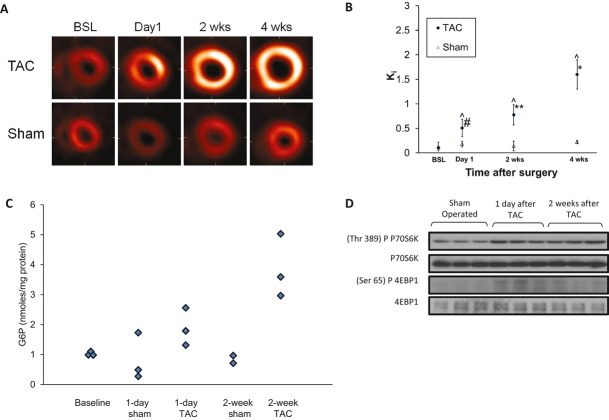
Metabolic remodeling and mTOR activation precede structural remodeling in hearts subjected to high workload in vivo. A, Representative serial transverse, end‐diastolic PET slices for TAC and sham‐operated mice 1 day, 2 weeks, and 4 weeks after surgery. One day after TAC, there was an increase in FDG uptake that increased further over 4 weeks. B, Quantification of the rate of cardiac FDG uptake (K_i_) of PET images from all TAC (n=8) and sham‐operated (n=5) mice. Data shown are mean±SEM. K_i_ in TAC mice demonstrated a 5‐fold increase in FDG uptake on day 1 and a 1.5‐ to 3.2‐fold increase from day 1 to 4 weeks. Sham‐operated mice showed no significant change in FDG uptake over 4 weeks. Comparisons at different times within TAC group: day 1 vs baseline (BSL), #*P*<0.05; 2 weeks vs BSL or day 1, **P<0.05; 4 weeks vs BSL, day 1, or 2 weeks, *P<0.001. Comparisons between TAC and sham groups at the same points, ^*P*<0.05. C, Tissue G6P levels in hearts after TAC or sham operation at baseline and after 1 day and 2 weeks. Dot plots of G6P levels for each group; n=6 for TAC and n=5 for sham. G6P levels were 2.3‐ and 4.6‐fold higher compared with sham‐operated animals 1 day and 2 weeks after TAC, respectively. Kruskal–Wallis test yielded overall *P*=0.0356. D, Representative Western blots demonstrated an increase in p70S6K and 4EBP1 phosphorylation 1 day and 2 weeks after TAC. mTOR indicates mammalian target of rapamycin; PET, positron emission tomography; TAC, transverse aortic constriction; FDG, 2‐deoxy, 2[^18^F]fluorodeoxy‐glucose.

For Figure 8, we assessed whether metabolic changes observed in stressed hearts *ex vivo* could be observed in an in vivo setting. We used PET scan to assess glucose accumulation over time between sham‐operated (n=5) and TAC (n=8) mice using a Friedman test. We corroborated the findings from the imaging by comparing G6P levels in hearts of mice before surgery (n=3), 1 day post–sham operation (n=3), 1 day post‐TAC (n=3), 2 weeks post–sham operation (n=2), and 2 weeks post‐TAC (n=3) using a Kruskal–Wallis test.

**Figure 9.**
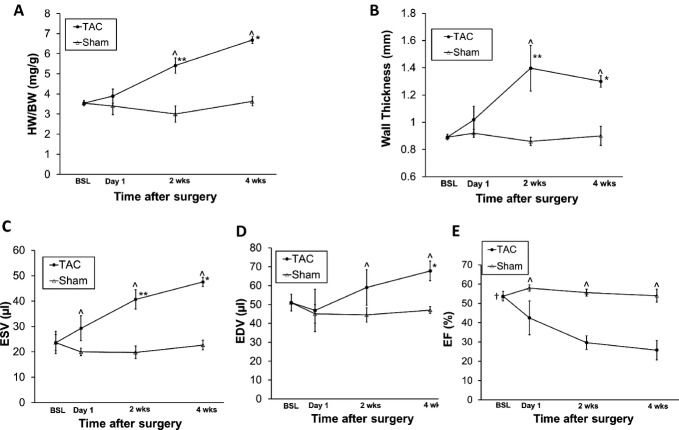
In vivo structural and functional changes accompany metabolic changes in response to pressure overload. Data shown are mean±SEM; n=8 for TAC and n=5 for shams. A, Ratio of heart weight (HW) measured using MRI and body weight (BW). HW/BW ratio remained unchanged 1 day after TAC, but increased by 1.4‐ and 1.7‐fold between day 1 and 4 weeks, respectively. HW/BW showed no significant change over 4 weeks in sham‐operated mice. B, End‐diastolic wall thickness measured in vivo using MRI. Wall thickness was unchanged 1 day after TAC, but increased about 1.4‐fold between day 1 and 4 weeks. Wall thickness showed no significant change over 4 weeks in sham‐operated mice. End‐systolic volume (ESV; C), end‐diastolic volume (EDV; D), and resultant ejection fraction (EF; E) assessed in vivo using MRI imaging. An increase in ESV and decline in EF occurred 1 day after TAC. Sham‐operated mice exhibited no significant change in EDV, ESV, and EF over 4 weeks. Comparisons at different times in the TAC group: day 1 vs baseline (BSL), #*P*<0.05; 2 weeks vs BSL or day 1, **P<0.05; 4 weeks vs BSL or day 1, *P<0.01; BSL vs day 1, 2 weeks, and 4 weeks, †*P*<0.05. Comparisons between TAC and sham groups at the same points, ^*P*<0.05. Two‐way repeated‐measures ANOVA analyzed, and Holm‐Sidak post hoc test performed to obtain individual significance factors. TAC indicates transverse aortic constriction; MRI, magnetic resonance imaging; ANOVA, analysis of variance.

For Figure 9 we compared sham‐operated (n=5) and TAC (n=8) mice at various times with 2‐way repeated‐measures ANOVA followed by a Holm–Sidak test to show the timing of structural changes in response to increased workload.

**Figure 10.**
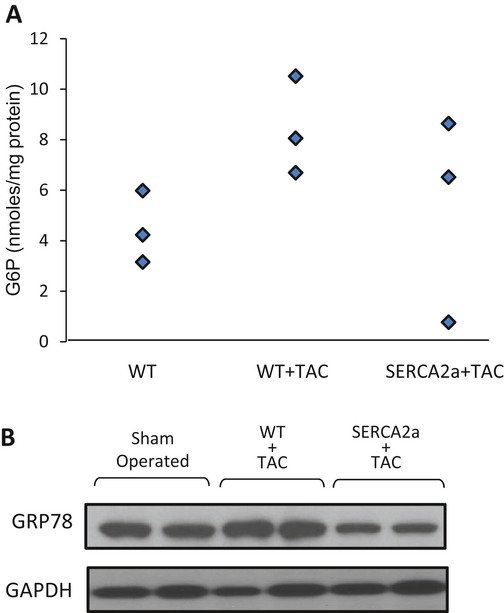
Cardiac‐specific SERCA2a overexpression reduces intracardiac G6P and decreases markers of ER stress in mice subjected to TAC in vivo. A, Effects of SERCA2a overexpression on tissue G6P levels in TAC mice. G6P levels were 1.9‐fold higher on average in WT+TAC than in WT−TAC. SERCA2a overexpression slightly reduced G6P accumulation in hearts subjected to TAC. Dot plot of G6P levels (n=3 for each group). Kruskal–Wallis test yielded an overall *P*=0.15. B, Representative Western blots demonstrated an increase in GRP78 protein expression in WT+TAC, which was reduced with SERCA2a overexpression (n=6 for each group). In vivo contractile performance, assessed by echocardiography, was improved in SERCA2a+TAC compared with WT+TAC (data not shown). ER indicates endoplasmic reticulum; WT, wild type; G6P, glucose 6‐phosphate; TAC, transverse aortic constriction.

For Figure 10 we evaluated the effects of ER stress relief on glucose metabolism by genetically overexpressing SERCA2a comparing G6P levels in wild type (n=6), wild type+TAC (n=6), and SERCA2a overexpression+TAC (n=6) using a Kruskal–Wallis test.

**Figure 11.**
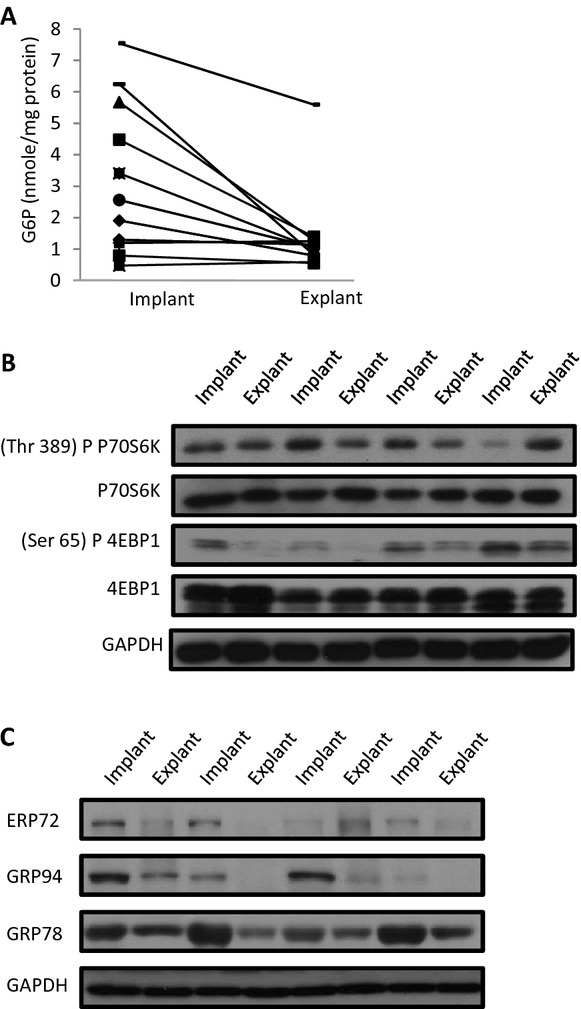
Mechanical unloading of failing human hearts results in reduced G6P accumulation, reduced mTOR activation, and reduced ER stress markers. A, Tissue G6P levels for individual patients with idiopathic dilated cardiomyopathy before and after mechanical unloading; n=11 paired samples, *P*<0.05 using paired *t* test. B, Representative Western blots of p70S6K and 4EBP1 before and after mechanical unloading. C, Representative (each “implant” and “explant” refers to 1 patient's heart—4 patients total) Western blots of markers of ER stress (ERP72, GRP94, GRP78) before and after mechanical unloading. G6P indicates glucose 6‐phosphate; ER, endoplasmic reticulum; mTOR, mammalian target of rapamycin.

For Figure 11, to determine whether the metabolic findings in animal models translated to findings in failing human hearts, we compared G6P levels of heart samples before and after mechanical unloading with an LVAD (n=11 pairs) using a paired *t* test.

Statistical analyses of all *ex vivo* data was performed using STATA v.10 (College Station, TX), whereas the remaining in vivo data were analyzed using SigmaStat 3.0 (SPSS, Inc, Chicago, IL). *P<*0.05 was considered significant for all analytic models.

Adjustments for multiple comparisons were not performed because (1) the data were exploratory in nature; and (2) although the experiments (and their associated statistical analyses) were performed to assess the associations between different independent and dependent variables, taken as a whole they are presented as evidence of our global hypothesis that stress and glucose accumulation are necessary to cause mTOR activation and expression of ER stress markers. Therefore, the results are interrelated because they are all describing different aspects of the same physiological phenomenon, and thus each experiment provides additional evidence (and strengthens our argument) for the overall hypothesis. Although there is a possibility of type I errors (false‐positive results), we draw the reader's attention to the critical evaluation of the results of each experiment in light of the results of the other experiments.

## Results

### mTOR Activation and Impaired Cardiac Power With Increased Workload

Irrespective of the substrate present in the perfusate, increased workload alone induced phosphorylation of PI3K, and Akt (at both Thr308 and Ser473; Figure 1A). However, strong phosphorylation of TSC2, mTOR, p70S6K, and 4EBP1 was observed only in the presence of glucose at high workload. Rapamycin pretreatment of the rats for 1 week inhibited phosphorylation of Akt (at both Thr308 and Ser473), TSC2, mTOR, p70S6K, and 4EBP1. mTOR signaling was not activated in hearts perfused either at normal workload with glucose or at increased workload in the absence of glucose.

To determine the functional consequence of mTOR activation, we measured cardiac power in hearts from vehicle‐treated and rapamycin‐treated rats perfused with glucose or NCS (Figure 1B). At normal workload, there was no significant difference in mean cardiac power between the experimental groups (n=5 to 7 per group). When subjected to increased workload, all perfused hearts demonstrated an increase in cardiac power relative to their normal workload values. However, in the vehicle‐pretreated group, hearts perfused with glucose as the only substrate did not increase their cardiac power to the same extent as the other 2 groups. Because this differential effect of glucose on cardiac power was mitigated by pretreatment with rapamycin, the difference is most likely mTOR dependent. To evaluate whether the above differences were the result of changes in efficiency, we also measured oxygen consumption in groups at comparable workloads (n=3 for each group; Figure 2A). There was no difference in oxygen consumption between groups at comparable workloads. Efficiency also did not change (n=3 for each group; Figure 2B), but it showed a tendency toward increase in the rapamycin‐treated group.

We also measured UCP3. Both mRNA (Figure 3A) and protein (Figure 3B) levels of UCP3 were unchanged with rapamycin treatment or increased workload, suggesting that mitochondrial efficiency was also unchanged with rapamycin pretreatment (2 to 3 repeated measures for each animal). We also noted that under all experimental conditions the adenine nucleotide levels (ATP, ADP, and AMP) did not differ significantly despite differences in mTOR activity and contractile function (data not presented). This is in keeping with our earlier observation that the levels of ATP, ADP, and AMP as well as total adenine nucleotides do not change with increased workload of the perfused heart.^17^

### Glucose 6‐Phosphate (G6P) and mTOR Activation

To elucidate possible mechanisms of glucose‐mediated mTOR activation, rates of glucose uptake and oxidation were simultaneously quantitated in *ex vivo* hearts (n=5 to 6 per group). At normal workload, rates of glucose uptake (4.2±0.7 μmol/min per gram dry weight) matched rates of glucose oxidation (3.9±0.7 μmol/min per gram dry weight) (Figure 4A). However, when the workload was increased, rates of glucose uptake (11.5±0.8 μmol/min per gram dry weight) exceeded rates of glucose oxidation (9.5±0.6 μmol/min per gram dry weight) by >10%. Rapamcyin pretreatment reduced rates of glucose uptake and rates of glucose, correcting the mismatch between glucose uptake and oxidation (Figure 4A). Lactate production, an indirect measure of glycolytic activity, was unchanged in both groups (data not shown).

Because mTOR activation was associated with a suggested mismatch between glucose uptake and oxidation, we investigated whether the accumulation of glucose or one of its metabolites was associated with mTOR activation (n=3 to 4 per group). G6P levels were 4‐fold higher on average in hearts subjected to an acute increase in workload (4.8±1.0 nmol/mg protein) compared with hearts perfused at normal workload (1.3±0.2 nmol/mg protein). Rapamycin pretreatment severely reduced the tissue content of G6P (0.36±0.1 nmol/mg protein) in hearts perfused with glucose at increased workload (Figure 4B). In contrast, levels of other glycolytic intermediates were unchanged at high workload (data not shown).

To assess whether hexose‐6‐phosphate accumulation leads to load‐induced mTOR activation, we next perfused 2 groups of hearts with either of the 2 glucose analogues: 3‐O‐methylglucose (which is transported in and out of the cardiomyocyte, but not metabolized), or 2‐deoxyglucose (which is taken up by the cardiomyocyte, phosphorylated, and trapped as a G6P analogue and not further degraded).^15^ 2‐Deoxyglucose, but not 3‐O‐methylglucose, increased phosphorylation of TSC2, mTOR, and its downstream targets in hearts perfused at high workload (Figures 4C and S1). mTOR and its downstream targets were not phosphorylated in hearts perfused with either glucose analogue at normal workload. These experiments provided strong evidence that hexose phosphate levels are associated with mTOR activation in hearts subjected to increased workload.

We also perfused hearts with a “physiological” mix of substrates (a combination of glucose, NCS, and the long‐chain fatty acid oleate) at both normal and high workloads to mimic more closely the metabolic milieu of hearts in vivo (n=2 to 3 per group; Figure 5). Indeed, although not quite statistically significant, hearts perfused with this “physiological” mix of substrate at high workload showed a 2.5‐fold increase on average in G6P levels (Figure 5A) and increased phosphorylation of both downstream targets of mTOR, p70S6K and 4EBP1 (Figure 5B) compared with hearts perfused with NCS, which is similar to hearts perfused with glucose alone.

### G6P‐Dependent mTOR Activation and AMPK Downregulation

Next we investigated the effect of enzyme 5′‐AMP activated protein kinase (AMPK). AMPK is upstream of TSC2 and regulates fuel supply and substrate metabolism in the heart. When phosphorylated at Thr172, AMPK phosphorylates and inhibits acetyl‐CoA carboxylase (ACC), which is also used as a marker of AMPK activation. AMPK also phosphorylates TSC2 (Ser1387) and RAPTOR (Ser792) to inhibit mTOR. Neither increased workload nor glucose alone was associated with a change in AMPK phosphorylation (Figures 6A and S2). However, perfusion at increased workload with glucose resulted in downregulation of phospho‐AMPK (Thr172), phospho‐ACC (Ser79), and phospho‐TSC2 (Ser1387). Pretreating animals with rapamycin prevented the downregulation of AMPK, ACC, and TSC2 phosphorylation with increased workload and glucose as the only substrate. RAPTOR (Ser792) phosphorylation was unchanged with workload or rapamycin treatment (Figures 6A and S2).

To determine the functional consequences of AMPK activation, hearts from rats pretreated with metformin (500 mg/kg per day IP) for 7 days underwent *ex vivo* working heart perfusions. Metformin restored cardiac power by 26% in hearts perfused with glucose substrate at increased workload (n=5 to 7 per group; Figure 6B); 2 times were statistically significant (denoted by an asterisk in the figure). To examine whether changes in glucose metabolism secondary to AMPK activation were associated with the restoration of cardiac power, rates of glucose uptake and oxidation were quantitated. Systemic metformin pretreatment did not change rates of glucose uptake (11.7±1.1 μmol/min per gram dry weight) in perfused hearts subjected to high workload, but it improved rates of glucose oxidation (11.0±0.7 μmol/min per gram dry weight) (n=5 to 6 per group; Figure 6C) and blunted G6P accumulation (2.1±0.1 nmol/mg protein) compared with vehicle‐pretreated animals (n=3 to 4 per group; Figure 6D). To further determine whether AMPK mediates glucose‐dependent mTOR activation, animals were treated with the AMPK activator metformin for 7 days (500 or 250 mg/kg per day IP) before perfusion with glucose at high workload. Systemic treatment with metformin prevented AMPK downregulation and inhibited mTOR activation in hearts perfused with glucose at high workload (Figures 6E and S3). Metformin treatment had no effect on mTOR phosphorylation or on cardiac power in hearts perfused at physiologic workload (data not shown). To circumvent systemic or off‐target effects of metformin, we also perfused hearts of untreated animals with buffer containing metformin. The perfusion of hearts with buffer containing metformin demonstrated that metformin prevented AMPK downregulation and inhibited mTOR activation in a dose‐dependent manner (Figures 6E and S3).

### Glucose‐Mediated mTOR Activation and ER Stress

Because of increasing evidence that dysregulated mTOR activation disrupts protein quality control by inducing ER stress response,^10^ we asked whether the induction of ER stress response could be indirectly regulated by G6P and could be responsible for the decline in cardiac power in hearts perfused at high workload. Transcription of the ER chaperones GRP78, GRP94, and ERp72 is increased when a cell responds to ER stress.^28^ Perfusion at normal workload with glucose as the only substrate or at high workload with NCS did not increase markers of ER stress ( n=2 to 3 with 2 to 3 repeated measures per animal; Figure 7A). Because perfusion at high workload with glucose induced no significant increases in GRP78, GRP94, and ERp72 mRNA expression (Figure 7A), we examined GRP78 protein levels (Figure 7B) and found increases in hearts perfused with glucose at high workload. Hearts perfused at normal workload with glucose plus thapsigargin, an inhibitor of SERCA2a and an inducer of ER stress response, were used as positive controls (Figure 7B). Because prolonged ER stress causes cellular damage and induces apoptosis, we also assessed levels of GADD153/CHOP, a marker of ER‐associated apoptosis, which was unchanged under all untreated conditions, suggesting the absence of apoptosis (Figures 7B and S4). Changes in GRP78, GRP94, and ERp72 mRNA expression and GRP78 protein level were reversed by either rapamycin or metformin pretreatment of animals when hearts were perfused with glucose. There were no statistically significant differences compared with hearts perfused with NCS at high workload or with glucose at normal workload (Figure 7B and S4). In addition to upregulating ER stress chaperones, mTOR‐dependent regulation of autophagy controls protein homeostasis in cardiomyocytes. To determine whether changes in autophagy occur dynamically in response to workload with or without glucose, we assessed the LC3‐II:LC3‐I ratio and beclin, markers of autophagy, in the perfused heart samples. We discovered that workload, glucose, or pretreatment with rapamycin did not significantly change protein levels of the LC3‐II:LC3‐I ratio or beclin (data not presented).

To assess more specifically whether ER stress contributed to the decline in cardiac power in hearts perfused with glucose at increased workload, we added an ER “stress‐relieving” agent, the histone deacetylase inhibitor 4‐phenylbutyrate (PBA). Although PBA did not reduce mRNA expression of GRP78, GRP94, and ERp72 (Figure 7A), it reduced the protein level of GRP78 (Figure 7B) and was associated with a 27% improvement in cardiac power in hearts perfused at increased workload with glucose (n=5 to 8 per group; Figure 7C). PBA had no effect on cardiac power at normal workload. PBA also had no effect on mTOR or p70S6K phosphorylation. PBA pretreatment did not affect G6P levels in hearts at increased workloads (n=3 to 4 per group; Figure 7D).

Finally, we performed microarray analyses to comprehensively delineate whether alternative mechanisms could contribute to the observed improvements in contractile function in stressed hearts perfused with glucose after rapamycin, metformin, or phenylbutyrate treatment. Cluster analysis showed no significant clusters of gene expressions similarly regulated by all 3 treatment groups (data not shown).

### Metabolic and Functional Remodeling in Response to Increased Workload In Vivo

We wondered whether the findings observed *ex vivo* correlate with hearts subjected to pressure overload in vivo. In mice subjected to aortic constrictions (TAC, n=8), transverse end‐diastolic mid‐ventricular positron emission tomography (PET) images indicated an increase in 2‐deoxy,2‐fluoro‐d‐glucose (FDG) uptake beginning 1 day after transverse aortic constriction (TAC), which increased further and was sustained over 4 weeks (Figure 8A). Sham‐operated animals (n=5) showed no change in FDG uptake over the same period. Quantitatively, a 5‐fold increase in the rate of myocardial FDG uptake, K_i_ (mL/min per gram), a specific measurement of metabolic remodeling, was observed 1 day after TAC using a 3‐compartment model (Figure 8B). The rate of FDG uptake continued to increase (1.5‐ to 3.2‐fold) from 1 day to 4 weeks. Calculated K_i_ at baseline (0.11±0.03) agreed well with the measured K_i_ (0.14±0.06) obtained from arterial blood samples used for input function estimation. No significant difference in K_i_ values was observed over 4 weeks in shams. In addition, we assessed G6P levels in these hearts 1 day and 2 weeks after TAC to corroborate the metabolic remodeling observed with PET imaging. We observed a slight increase in G6P levels 1 day after TAC, and the increase was more pronounced 2 weeks after TAC (n=6 TAC and n=5 sham; Figure 8C). To determine the association between the increased G6P levels and mTOR activation in vivo, we assessed G6P levels and mTOR signaling 1 day and 2 weeks after TAC. Representative Western blots demonstrated an apparent increase in p70S6K and 4EBP1 phosphorylation at both times (Figure 8B).

Although significant change in metabolic remodeling was observed starting 1 day after TAC, there was no appreciable change at this early time in the heart weight to body weight ratio (HW/BW; Figure 9A). Table 3 summarizes additional metabolic, functional, and structural parameters measured 1 day after TAC. On day 1 there was a small increase in wall thickness. However, the increase in left ventricular pressure was accompanied by a much greater increase in the rate of glucose uptake (Figure 8A) and a decrease in the ejection fraction (Figure 9D). Thus, profound changes in cardiac metabolism were accompanied by functional changes (decreased ejection fraction), whereas the structural changes were small. Two and 4 weeks after TAC, the HW/BW had increased by 1.4‐ and 1.7‐fold, respectively. Similarly, end‐diastolic wall thickness increased significantly. No significant changes in HW/BW or wall thickness were observed over the same period in sham‐operated mice.

**Table 3.** Comparison of Baseline and Day 1 Values for Different Parameters Among TAC Mice

Parameter	Baseline	Day 1	Change (%)	n	*P* Value
K_i_	0.10	0.51	407.67	8	0.011
Ejection fraction, %	53.77	42.5	−20.9	8	0.011
HW/BW	3.54	3.89	9.89	5	NS
Wall thickness, mm	0.9	1.0	11.1	5	0.043
Heart rate, bpm	406.8	470.8	15.7	5	NS
LV systolic pressure, mm Hg	83.95	119	41.8	3	NS

*P* values were obtained using the Wilcoxon signed rank test. Change (%) indicates average change from baseline to day 1 in each of the measured corresponding parameters; “n” indicates number of animals used in each of the measured corresponding parameters; TAC, transverse aortic constriction; HW/BM, heart weight to body weight ratio; LV, left ventricle.

To see whether functional changes accompany metabolic changes and changes in cell signaling after TAC, end‐diastolic volume (EDV), end‐systolic volume (ESV) and ejection fraction (EF) were followed for 4 weeks. Increases in the ESV (Figure 9C) and significant decreases in the EF (Figure 9E) followed the metabolic changes seen 1 day after TAC. Both the ESV and EDV (Figure 10C and 10D) progressively increased 2 and 4 weeks after TAC. Subsequently, the EF continued to decrease 1.4‐ and 1.6‐fold 2 and 4 weeks after TAC, respectively. No significant changes in the EDV, ESV, or EF were observed in sham‐operated animals over 4 weeks. Taken together, the in vivo imaging experiments support the hypothesis that metabolic, signaling, and functional changes precede or accompany structural changes in pressure overload‐induced left ventricular hypertrophy (LVH).

Because increased expression of SERCA2a results in increased oxidation of glucose,^29^ we deployed a genetic model of SERCA2a overexpression to gain insight into the role of G6P in pressure overload–induced LVH. We subjected this cardiac‐specific SERCA2a overexpression mouse model to aortic constriction and measured G6P levels and ER stress markers in the heart. SERCA2a overexpression was associated with a slight reduction in G6P accumulation (not significant; Figure 10A) and a significant reduction in GRP78 protein expression (Figure 10B), suggesting that a tighter match exists between glucose uptake and oxidation and reduced ER stress in this model.

### Failing Human Hearts Before and After Unloading With a Left Ventricular Assist Device

Finally, we wished to assess whether the findings in rodent heart had any relevance to the failing human hearts of patients with nonischemic idiopathic dilated cardiomyopathy receiving a left ventricular assist device to unload the heart as bridge to transplant. Changes in energy substrate metabolism and ER stress have been observed in failing human hearts,^11,30^ but the effect of mechanical unloading on ER stress is not known. We noted that G6P levels dramatically decreased after mechanical unloading (Figure 11A), and to investigate whether changes in cardiac glucose metabolism accompanied changes in mTOR activation or ER stress, we assessed the downstream targets of mTOR (p70S6K and 4EBP1) and ER chaperones (GRP78, GRP94, ERp72). Phosphorylation of p70S6K and 4EBP1 (Figure 11B) and protein levels of GRP78, GRP94, and ERp72 (Figure 11C) all decreased after mechanical unloading with an LVAD, suggesting that tight coupling of glucose uptake and oxidation with mechanical unloading led to decreased mTOR activity and ER stress reduction.

## Discussion

**Figure 12.**
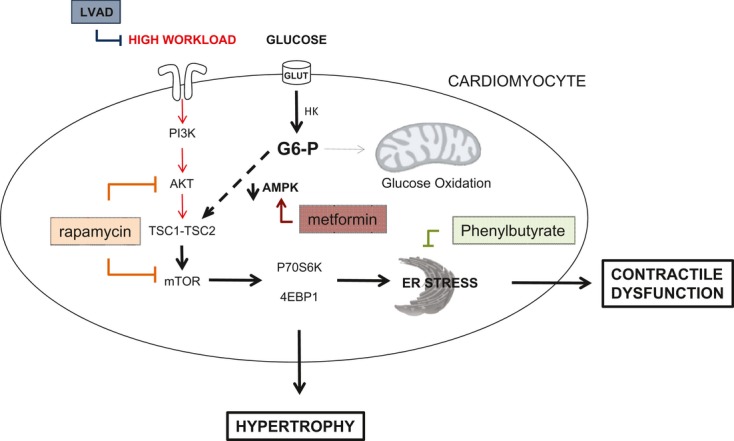
Proposed mechanism by which G6P accumulation regulates load‐induced mTOR activation and ER stress. The intersections of the metabolic pathway of glucose transport and phosphorylation with the molecular signaling pathways addressed in the study are shown. We propose that rapamycin (mTOR inhibition), metformin (AMPK activation), phenylbutyrate (ER stress relief), or LVAD (mechanical unloading) protect the heart from metabolic stress at high workload. G6P indicates glucose‐6‐phosphate; mTOR, mammalian target of rapamycin; ER, endoplasmic reticulum; AMPK, AMP kinase; LVAD, left ventricular assist device; HK, hexokinase; TSC, or tuberous sclerosis complex, is composed of TSC1 (hamartin) and TSC2 (tuberin).

We set out to test the hypothesis that a metabolic signal links hemodynamic stress with activation of the mTOR pathway and ER stress response. Our main findings are summarized in Figure 12 and include the following 6 points. (1) Doubling the workload of rat hearts perfused *ex vivo* with glucose as the only substrate increases rates of myocardial glucose uptake beyond the hearts' oxidative capacity, resulting in G6P accumulation, which mediates load‐induced mTOR activation. (2) Load‐induced mTOR activation impairs cardiac power and induces ER stress response in a glucose‐dependent manner. Administration of rapamycin or metformin in vivo or directly relieving ER stress by 4‐phenylbutyrate rescues cardiac power. (3) Correcting the mismatch between glucose uptake and oxidation in the stressed heart *ex vivo* and in vivo prevents G6P accumulation, relieves the ER stress response, and rescues contractile function in hearts subjected to pressure overload. (4) In hearts subjected to increased workload in vivo, metabolic remodeling precedes structural remodeling of the heart and accompanies load‐induced contractile dysfunction. (5) When pressure overload is imposed on the heart in a transgenic mouse model of increased contractility, contractile function is preserved in the absence of the ER stress response. (6) In the failing human heart, mechanical unloading decreases levels of G6P, reduces mTOR activation, and relieves ER stress.

These findings deserve consideration in a broader context. Although the normal heart preferentially oxidizes fatty acids, the increased energy requirements of hearts subjected to high workload are met by the oxidation of carbohydrates.^1^ The classic explanation for this phenomenon is based on the hypothesis that the heart oxidizes the most efficient substrate for a given environment.^13^ Aside from merely providing energy for cell function, however, glucose metabolites also regulate gene expressions in the liver, pancreatic β‐cells,^31–32^ and heart.^33^ More recently, we have suggested that the glucose metabolite G6P mediates activation of the transcription factor carbohydrate responsive binding protein (ChrebP)^34^ and that G6P activates insulin‐dependent mTOR signaling in the unstressed heart.^5^ We now provide evidence in 3 models (mouse, rat, and human heart) to suggest that load‐induced mTOR activation and the ensuing ER stress are mediated by G6P, as well.

Until now, no study has addressed whether cardiac substrate metabolism can regulate “load‐induced” hypertrophy via the mTOR signaling pathway.^8,35–36^ Our findings in the working heart *ex vivo* demonstrate that acute increases in workload require glucose metabolism to activate mTOR, suggesting that “load‐induced” hypertrophic signaling through mTORC1 is substrate specific. More to the point, we propose that mTORC1 is activated by the metabolic signal G6P, which accumulates at increased workload when rates of glucose uptake exceed the heart's oxidative capacity for glucose. The G6P hypothesis is confirmed by the glucose analogue 2‐deoxyglucose, which is phosphorylated to 2‐deoxyglucose 6‐phosphate and not metabolized further, resulting in strong activation of mTOR (Figure 4C). In contrast, the glucose analogue 3‐O‐methylglucose, which is transported into the cell and not phosphorylated, exhibited no effect on mTOR activation (Figure 4C). We consider this evidence compelling and in support of our hypothesis.

The metabolic findings require a discussion of mTOR activation by a metabolic signal. In the unstimulated state, mTORC1 is inhibited by the heterodimer tuberous sclerosis complex (TSC), composed of hamartin (TSC1) and tuberin (TSC2). Differential phosphorylation states of TSC2 are necessary to dissociate the complex to release its inhibition on mTORC1. We dissected the load‐ and substrate dependence of mTORC1 signaling in the heart and determined that increased workload alone triggers PI3K, Akt, and minimal Akt‐dependent phosphorylation of TSC2 at Ser939, regardless of glucose. However, complete load‐induced mTORC1 activation requires downregulation of AMPK‐dependent phosphorylation of TSC2 at Ser1387, which occurs with G6P accumulation. Indeed, AMPK activation with metformin, both in vivo and *ex vivo*, prevents G6P‐mediated mTOR activation at increased workload. In skeletal muscle, high levels of glucose activate mTOR in an AMPK‐dependent manner by modulating the redox state.^37^ In heart, the redox state does not change with workload.^17^ However, like in skeletal muscle, AMPK downregulation by glucose in the heart occurs independently of the energy state, which is consistent with previous work both *ex vivo*^17^ and in vivo.^38^ In the genetically modified ACSL1−/− mouse, in which cardiac glucose metabolism is upregulated (like a heart subjected to increased workload), mTOR activation correlates with G6P accumulation and AMPK downregulation independent of change in the AMP/ATP ratio.^39^ Taken together, our study reveals that load‐induced mTOR activation is mediated by changes in glucose metabolism.

Excessive glucose uptake by the heart has been associated with contractile dysfunction and deemed glucotoxic by increasing flux through the hexosamine biosynthetic pathway, dysregulating protein glycosylation, and producing reactive oxygen species.^40–41^ Genetically modified mice that take up excess glucose but also demonstrate enhanced glycolytic flux are, however, protected from glucotoxicity.^42–43^ We now propose that intermediary glucose metabolite (G6P) accumulation itself may impose metabolic stress on the heart and contribute to contractile dysfunction by activating mTOR and inducing endoplasmic reticulum (ER) stress.

Sustained mTOR activation synthesizes proteins beyond the folding capacity of the ER and induces ER stress.^28^ By evaluating ER chaperone expression as an early marker of ER stress, we demonstrate that G6P accumulation impairs contractile function by inducing the ER stress response. Although the mechanism by which ER stress causes contractile dysfunction has yet to be defined, we suspect that it involves a perturbation of calcium handling by the cardiomyocyte. Indeed, contractile dysfunction in isolated rodent hearts perfused with high concentrations of glucose is associated with the presence of abnormal calcium transients.^44^ Because the ER regulates calcium flux and excitation–contraction coupling, it is conceivable that G6P‐mediated ER stress is responsible for impairing contractile function by inducing abnormal calcium transients. Consistent with our hypothesis, cardiac‐specific SERCA2a‐overexpressing mice demonstrate a reduction in markers of ER stress when subjected to pressure overload in vivo. Specifically, we suggest a critical role for ER chaperone GRP78, which is involved in calcium transport from the ER to the mitochondria.^45^ In our studies, direct relief of ER stress with PBA rescued contractile function in hearts perfused with glucose at high workload. PBA is already approved by the US Food and Drug Administration for treating urea cycle disorders and currently used in clinical trials to treat cystic fibrosis.^46^ It may have therapeutic benefit in load‐induced heart disease as well.

Rapamycin pretreatment also rescues G6P‐mediated ER stress and cardiac dysfunction at increased workload. Rapamycin binds and disrupts the mTOR complexes. We now discover that its indirect metabolic effects may mediate via its inhibition of mTOR. Prolonged rapamycin treatment inhibits mTORC2 assembly, which prevents phosphorylation of Akt,^47^ a known regulator of glucose uptake in the heart.^48^ Therefore, not surprisingly, we observed that rapamycin administration blunts Akt phosphorylation and rates of glucose uptake at increased workload. In doing so, rapamycin prevents the mismatch between glucose uptake and oxidation, depletes intracardiac G6P, inhibits mTOR, and relieves ER stress.

Despite lowering rates of myocardial glucose uptake and oxidation to “unstressed” levels, rapamycin treatment also improves cardiac power (Figures 1 and 4). From an energetic perspective, this suggests that the increased energy requirements of hearts subjected to high workload, which are met by the oxidation of carbohydrates, may largely be responsible for fueling protein turnover and protein quality control (“internal work”). Protein turnover utilizes roughly 15% to 20% of a cell's energy in the resting state^49^ and at least 3 times more energy during times of growth.^50–51^ It is, therefore, tempting to speculate that the energy conserved by rapamycin's inhibition of protein synthesis and ER stress improves cardiac efficiency and contributes to its ability to reverse load‐induced cardiac dysfunction.^8,35,52^ Furthermore, the improvement in cardiac power observed in rapamycin‐treated rats gives credence to the hypothesis that the hypertrophic process may not be necessary to maintain systolic function in hearts subjected to increased workload.^53^ Our results offer a new perspective on the energy cost of protein synthesis and protein quality control.

Metformin inhibits ER stress in isolated mouse aortas from mice fed an athrogenic diet^54^ and in cardiomyocytes subjected to ER stress by reperfusion after hypoxia.^55^ Although metformin is known to improve cardiac function in murine models of heart failure,^56–57^ its ability to alleviate ER stress in hearts subjected to increased workload has yet to be studied. We have demonstrated that metformin inhibits G6P‐mediated mTOR activation in a dose‐dependent manner and protects against load‐induced ER stress. When administered systemically, metformin also improves the oxidative capacity of the heart, blunts G6P accumulation, and improves cardiac function. Therefore, we propose that, like rapamycin, metformin metabolically protects hearts subjected to increased workload and, in doing so, may account for improving survival in diabetic patients with heart failure.^58^

Indeed, markers of ER stress are also upregulated in hypertrophied and failing heart after aortic constriction.^11^ We now report that in hearts from a cohort of 11 nondiabetic patients with idiopathic dilated cardiomyopathy and heart failure, mechanical unloading with an LVAD reduces intracardiac G6P levels and decreases mTOR activation and protein levels of ER stress markers. We observed a significant decrease in G6P accumulation after LVAD support. We have previously observed decreased myocardial glycogen content in failing human hearts after mechanical unloading,^59^ and more recently, proteomic analysis has revealed upregulation of proteins involved in glycolysis, energy, and oxidative metabolism in LVAD‐supported patients.^60^ Taken together, these studies support our findings and suggest that mechanical unloading promotes recoupling of glucose uptake and oxidation, prevents intermediary metabolite accumulation, and conserves energy in the failing heart by reducing ER stress. However, we wish to emphasize that we relied on metabolic and/or pharmacologic interventions in our experimental work. The results are therefore largely descriptive, and off‐target effects cannot be discounted.

Finally, previous studies performed in rat models of progressive hypertrophy used the tracer FDG analogue and semiquantitative measurements of myocardial FDG standardized uptake value with partial volume corrections.^61^ We have shown quantitatively in vivo that enhanced glucose uptake in the heart accompanies load‐induced contractile dysfunction before an increase in left ventricular mass. When increased, workload was sustained for up to 4 weeks, and increased wall thickness and HW/BW ratio directly correlated with increased FDG retention, suggesting that metabolic remodeling precedes and sustains structural remodeling of the heart.

In summary, we have provided evidence in support of the concept that metabolic remodeling precedes, triggers, and sustains structural remodeling of the heart. Specifically, we propose that dysregulated glucose metabolism and subsequent G6P accumulation mediate load‐induced mTOR activation, ER stress, and contractile dysfunction. Our study highlights the general notion that intermediary metabolism is a rich source of signals for cardiac growth and demonstrates the potential to reduce internal work and improve cardiac efficiency by targeting the metabolic axis in load‐induced heart disease.

## Disclosure

None.
